# Designing and Implementing a Simulation-Based Pediatric Trauma Training Program in a Resource-Limited Setting: The PRACTICE Study

**DOI:** 10.3390/pediatric18040109

**Published:** 2026-08-06

**Authors:** Jakob Olbrich, Alexander Hönning, Icaro Luan Tavares Latado, Andrea Laufer, Janna Schwab, Erik Haucke, Alexander Jünemann, Uwe Weibrecht, José de Ribamar Bandeira Filho, Kristina Zappel, Sinan Bakir

**Affiliations:** 1 Center for Emergency Training (ZfN), BG Klinikum Unfallkrankenhaus Berlin, 12683 Berlin, Germany; icaroluan.tavareslatado@ukb.de (I.L.T.L.); erik.haucke@ukb.de (E.H.); alexander.juenemann@ukb.de (A.J.); 2 Center for Orthopaedics, Trauma Surgery and Rehabilitation Medicine, University Medicine Greifswald, 17475 Greifswald, Germany; 3 Center for Clinical Research (ZkF), BG Klinikum Unfallkrankenhaus Berlin, 12683 Berlin, Germany; 4 Clinic for Anesthesiology, Critical Care Medicine, and Pain Management, BG Klinikum Unfallkrankenhaus Berlin, 12683 Berlin, Germany; 5 Clinic for General Orthopedics and Tumor Orthopedics, Muenster University Hospital, 48149 Muenster, Germany; andrea.laufer@ukmuenster.de; 6 Department of Trauma Surgery, Hand Surgery, and Sports Medicine, Vivantes-Humboldt Klinikum, 13509 Berlin, Germany; 7 Associação ProBrasil Nordeste, Coronel José Dias 64793-000, PI, Brazil; uwe.weibrecht@probrasil-nordeste.org; 8 Lucídio Portella Children’s Hospital, Teresina 64001-450, PI, Brazil; 9 Robert Koch Institute, 13353 Berlin, Germany; 10 Center for Orthopedics and Trauma Surgery, Helios Hospital Berlin-Buch, 13125 Berlin, Germany; 11 Medical School Berlin (MSB), University of Health and Medicine, 14197 Berlin, Germany

**Keywords:** traffic accident, pediatric trauma, crew resource management, middle-income countries, trauma training, simulation-based education, global health, Brazil, simulation training, emergency medicine

## Abstract

Background/Objectives: Road traffic accidents represent the leading cause of death in children and adolescents in Brazil. In the state of Piauí, non-specialized facilities frequently manage critically injured pediatric patients. Targeted training programs are considered a key strategy for improving outcomes. This study assessed the feasibility of adapting a pediatric trauma course developed in a high-income setting for medical first responders in both pre-hospital and in-hospital settings in a resource-limited environment in northeastern Brazil. Methods: This prospective non-randomized mixed-methods feasibility-oriented implementation and educational evaluation study involved the design of a simulation-based course based on a literature review by a German interprofessional and interdisciplinary team. Local adaptation was achieved through a needs assessment, field visits, and stakeholder collaboration. Implementation included the training of Brazilian instructor candidates, who subsequently delivered the course under supervision. Data were collected using study-specific, non-validated questionnaires at two timepoints and analyzed descriptively and exploratively. Results: A total of 98 healthcare professionals participated in the needs assessment (mean experience 9.7 ± 6.3 years; 62.2% nursing staff); 66 completed the pediatric trauma management section. Although familiarity with the ABCDE approach was high (86%), confidence in pediatric trauma management was significantly lower than management in adults (47% vs. 68%, *p* = 0.0018), particularly for invasive procedures. During implementation, 38 participants completed the course, with a 100% recommendation rate; approximately 80% felt prepared to teach independently. However, participants and instructors highlighted the need for more practical training, longer course duration, and follow-up instructor training. Conclusions: A context-adapted, simulation-based pediatric trauma training program was feasibly implemented and well accepted in a resource-limited region of Brazil. The train-the-trainer approach shows promise for strengthening local pediatric trauma capacity, but sustained implementation requires continued instructor development, supervised teaching, and long-term evaluation of educational and clinical outcomes.

## 1. Introduction

Unintentional injuries remain a major cause of death and disability among children and adolescents worldwide. According to the World Report on Child Injury Prevention, more than 830,000 children die each year as a result of preventable injuries [1]. In Brazil, road traffic injuries represent the leading cause of death among children and adolescents and contribute substantially to long-term disability, placing a considerable burden on affected individuals, families, and healthcare systems [1,2,3].

This burden is particularly relevant in the northeastern state of Piauí, where traffic-related mortality has increased markedly over recent decades, largely driven by motorcycle-related injuries [4]. In this predominantly rural region, critically injured children are frequently managed in non-specialized healthcare facilities because of long transport distances, limited access to pediatric referral centers, and constrained pre-hospital and emergency care resources [5,6]. As a result, healthcare professionals working in pre-hospital emergency medical services (SAMU) and emergency care units (UPAs) are often required to provide initial stabilization of severely injured pediatric patients under resource-limited conditions.

Previous studies have demonstrated that pediatric trauma care in low- and middle-income countries (LMICs) is frequently limited by insufficient training opportunities, lack of pediatric-specific expertise, and the absence of context-adapted clinical protocols [7,8,9,10,11,12]. These challenges are particularly relevant because critically ill and injured children are encountered relatively infrequently, making it difficult for healthcare professionals to acquire and maintain competence in high-risk, low-frequency procedures. Standardized educational programs, simulation-based training, and protocolized approaches have therefore been identified as important strategies for improving pediatric emergency care and reducing preventable mortality [1,8,9,10,11,12,13].

Despite the availability of internationally established training programs, such as Pediatric Advanced Life Support (PALS^®^), Advanced Trauma Life Support (ATLS^®^), and Pre-hospital Trauma Life Support (PHTLS^®^), important barriers limit their applicability in many low- and middle-income countries. High course costs, language requirements, resource-intensive training formats, and dependence on healthcare infrastructure that may not be available in remote settings restrict widespread implementation. In addition, neither program fully addresses the specific challenges of pediatric trauma care in resource-constrained environments, as PALS^®^ focuses primarily on pediatric medical emergencies, and ATLS^®^ and PHTLS^®^ are largely oriented toward adult trauma. These limitations highlight the need for context-adapted, resource-sensitive training programs that address local epidemiology, healthcare structures, and available treatment options while preserving the core principles of evidence-based trauma care.

Within the PRACTICE project (PReventing And Customizing Treatment of road traffic associated Injuries in ChildrEn), a context-adapted pediatric trauma training program was developed through collaboration between German and Brazilian partners. The objective of the present study was to evaluate the feasibility of adapting and implementing a simulation-based pediatric trauma course originally developed in a high-income healthcare setting for use in a resource-limited region of northeastern Brazil and to assess its acceptance among local healthcare professionals.

## 2. Materials and Methods

### 2.1. Study Overview

PRACTICE was designed as a funded, two-year international collaborative initiative comprising two components: (1) the design and implementation of a standardized training course for the management of critically injured children and adolescents, and (2) preventive interventions aimed at increasing awareness of traffic-related risks among young schoolchildren and their families. While the present study focuses exclusively on the conception, adaption and implementation of the training component, the follow-up and the prevention component were equally important in the project and will be reported in a separate publication to ensure thematic consistency and readability.

The PRACTICE project officially commenced on 15 August 2023. While formal funding approval was issued in November 2023, the funding period applied retrospectively from the project start, thereby covering the conception phase and the initial field visit.

The **conception phase** was conducted from 1 September 2023 to 31 January 2024, followed by an **implementation phase** from 1 February to 14 March 2024 (Table 1).

### 2.2. Conception Phase

#### 2.2.1. German Course Team Formation

The development of the PRACTICE course was led by the Center for Emergency Training, Department of Unfallkrankenhaus Berlin. To ensure interprofessional and interdisciplinary input, the German team consisted of five members: one internal medicine specialist with specialization in emergency medicine and intensive care, one orthopedic/trauma surgery resident with specialization in emergency medicine, one paramedic, and two registered nurses. Four team members were certified Crew Resource Management (CRM) instructors, two were active Pediatric Advanced Life Support (PALS^®^) instructors, and one held active certification as a Tactical Emergency Casualty Care (TECC^®^) instructor.

#### 2.2.2. Brazilian Partners Engagement and Field Visits

The non-governmental organization (NGO) ProBrasil served as the coordinating partner, facilitating continuous communication between all bilateral stakeholders.

The project was conducted in the Serra da Capivara region, Piauí, Brazil. Local partners in São Raimundo Nonato included the emergency care unit (UPA), the mobile emergency medical service (SAMU) and the municipal administration. In Teresina, collaborative partners were the State University of Piauí (UESPI), Lucídio Portella Children’s Hospital, and the state fire brigade (CBMPI).

An initial seven-day field visit, including observational clinical shifts in UPA and SAMU, provided insights into local infrastructure, staff qualifications, and clinical workflows.

Subsequently, six bilateral online meetings were conducted to adapt the course to local requirements. These included focus group discussions and qualitative interviews addressing feasibility, content, target groups, potential challenges, and identification of suitable training sites and future instructors, named multiplicadores. Preference was given to long-term staff as multiplicadores to mitigate the effects of workforce turnover. No formal instructor selection criteria were defined prospectively, and previous teaching experience was not systematically recorded.

Updated inventories of available equipment and medications were continuously provided to support context-specific course development, which served primarily as needs-assessment and curriculum-development activities. Written meeting protocols were maintained throughout the project; however, these discussions were not conducted as formal qualitative research interviews and were therefore neither audio-recorded nor subjected to a validated analysis.

#### 2.2.3. Course Design and Manual

Course development was informed by a literature review of established training programs, including PALS^®^, ATLS^®^, and PHTLS^®^, as well as pediatric trauma guidelines meeting AGREE-II quality criteria [14]. Based on this review, the curriculum (Table 2) was structured around four core components: (1) priority-based assessment; (2) essential interventional skills; (3) application of standard operating procedures (SOPs) for high-stress situations; and (4) integration of non-technical skills based on Crew Resource Management (CRM) principles.

The course emphasized the first hour of care in resource-limited settings and followed a priority-based approach using the ABCDE framework (Airway, Breathing, Circulation, Disability, and Exposure), an internationally established algorithm for the rapid assessment and prioritization of life-threatening conditions in critically ill and injured patients. Pediatric-specific adaptations were incorporated through the Pediatric Assessment Triangle (PAT), a rapid observational assessment tool for the initial evaluation of critically ill or injured children. To reflect this approach, the course material was organized into modules corresponding to the ABCDE sequence (“AB”, “C”, “DE”, plus “x” for catastrophic hemorrhage). The curriculum covered pediatric basic life support, hemorrhage control, airway and respiratory management (including supraglottic airway devices and needle decompression), circulatory support (including intraosseous access and tranexamic acid), and simplified analgesia. SOPs were incorporated into a structured course manual supported by age-adapted reference tables for vital signs, equipment sizes, and medication dosing. These SOPs were not intended to replace existing Brazilian Ministry of Health or SAMU protocols. Instead, they were reviewed together with local stakeholders and adapted to the locally available infrastructure, equipment, medications, and operational realities. The manual was designed in a portable DIN A5 format for both print and digital use.

Simulation-based scenarios with structured debriefings were used to integrate technical and non-technical competencies. CRM principles were incorporated to strengthen communication, situational awareness, leadership, and team performance. Following iterative review with local stakeholders, the final course manual was adapted to the local context and formally approved by the Brazilian project partners prior to implementation.

### 2.3. Data Collection, Course Rollout and Analysis

Data collection was conducted at two timepoints:

− Among local healthcare personnel in December 2023 and January 2024, and additionally during the local emergency medicine congress in Teresina, Piauí on 29 February 2024 (*needs assessment, timepoint 1*).
− Among course participants and the German instructor team after course delivery and completion, 17–28 February 2024 (*implementation phase evaluation, timepoint 2*).

**Figure 1:**
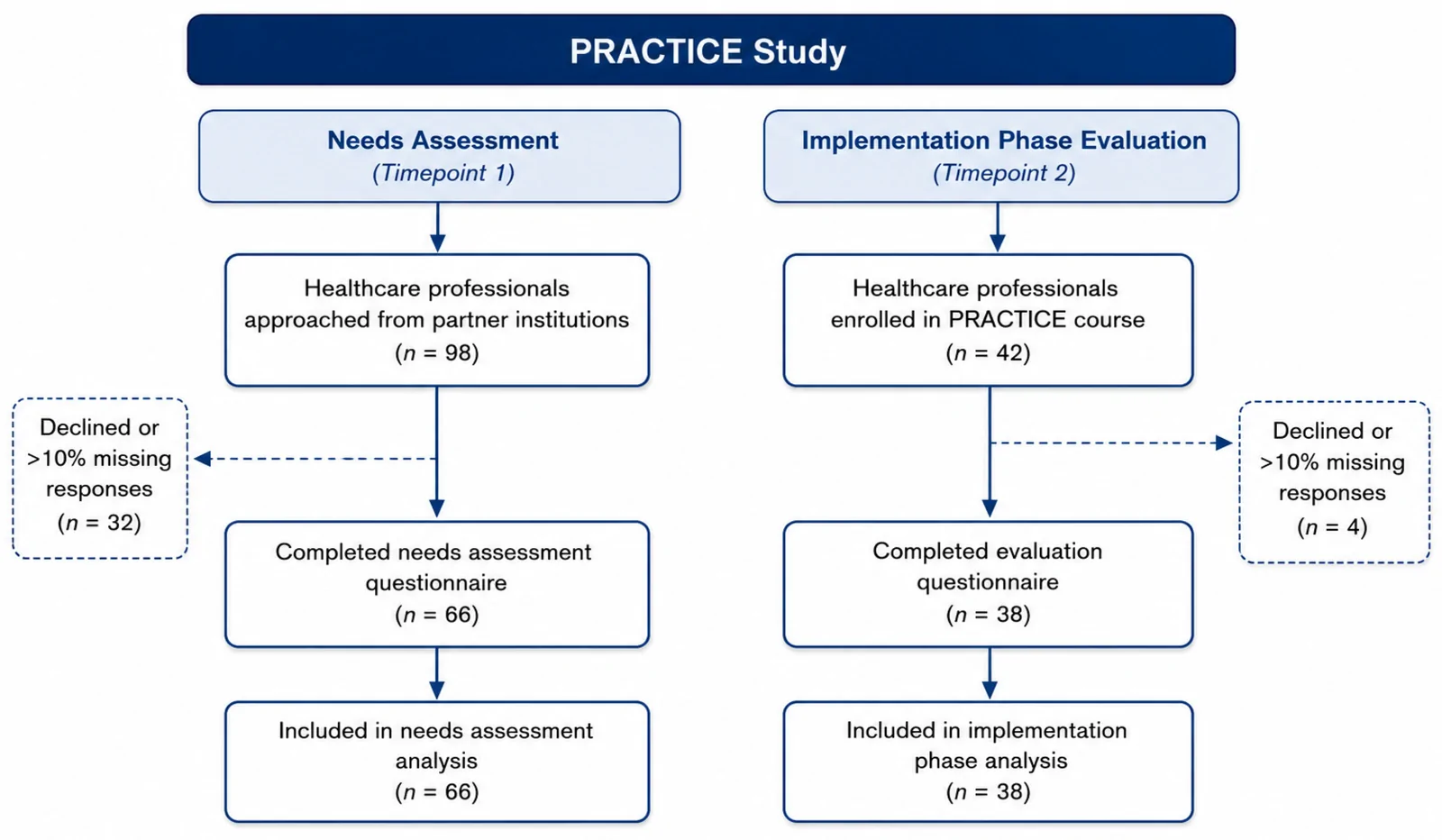
Participant flow of the PRACTICE study showing recruitment, questionnaire completion, exclusions, and inclusion in the analyses at both study timepoints.

Brazilian participants were recruited through local partner institutions. The flow of participants throughout the study is summarized in Figure 1.

Participation in the questionnaires was voluntary. Prior to enrolment, all participants received written participant information in Portuguese describing the project objectives, the educational intervention, the accompanying surveys, the pseudonymized processing of study data, and the intended scientific dissemination of anonymized project findings. Participants provided written informed consent before participation and data collection, including consent to publication. The primary purpose of the data collection during the conception and implementation phases was to inform local adaptation, implementation, and educational evaluation of the training course. The collected data were subsequently analyzed for scientific publication following completion of the implementation phase, after retrospective ethical approval had been obtained from the Ethics Committee of the University Medicine Greifswald. Prior to publication, the study procedures underwent an additional local review by the State Health Secretariat of Piauí (SESAPI), Brazil, which confirmed that no ethical concerns were identified and that the data collection complied with the applicable ethical, legal and regulatory requirements in Brazil.

All participants received the intervention. Blinding was not feasible owing to the educational nature of the intervention. No adverse events related to the questionnaire or educational intervention were observed.

The needs assessment aimed to identify training requirements, while the implementation phase evaluation focused on course acceptance, content, and organizational aspects. Since our primary objective was to assess the feasibility and practicality of the course, we used study-specific questionnaires to obtain targeted feedback from participants and instructors. These questionnaires were designed to address aspects that were not adequately covered by existing validated instruments.

As no suitable validated instruments addressing the specific objectives of the PRACTICE project were available, study-specific questionnaires were developed by the multidisciplinary German project team based on the educational objectives of the curriculum, relevant trauma guidelines, and previous experience in simulation-based medical education. The questionnaires subsequently underwent iterative review by the Brazilian project partners to ensure clarity, face validity, and contextual appropriateness prior to implementation. No formal psychometric validation or pilot testing was performed.

The evaluation was designed to assess feasibility, participant acceptance, and self-perceived preparedness using study-specific questionnaires. Objective assessments of knowledge or practical competence, such as written examinations or objective structured clinical examinations (OSCEs), were not included in the study design.

A three-day on-site training was conducted in Serra da Capivara, supported by ProBrasil and medical students from UESPI. On day one, the German instructor team trained eight Brazilian instructor candidates (multiplicadores). On days two and three, these multiplicadores delivered the course to local healthcare professionals under supervision.

Data were collected using standardized, non-validated online questionnaires administered via the GDPR-compliant platform LamaPoll. Responses were recorded on a five-point Likert scale ranging from “strongly disagree” to “strongly agree.”

Descriptive statistics included absolute (*n*) and relative (%) frequencies, arithmetic mean values (mean), standard deviation (sd) and ranges. Because this was an exploratory feasibility study, no formal prior sample size calculation was performed. Group comparisons for ordinal variables were performed using the Mann–Whitney U test for independent samples and the Wilcoxon signed-rank test for dependent samples, both at a significance level of α = 0.05. Given the exploratory design, results should be interpreted cautiously. Statistical analyses were conducted using STATA for Windows (StataCorp LLC, College Station, TX, USA), version 16.1.

## 3. Results

### 3.1. Course Adaptations Resulting from the Needs Assessment and Stakeholder Consultation

The needs assessment, observational shifts in SAMU and UPA, and repeated consultations with local stakeholders identified several contextual factors that differed substantially from the assumptions underlying established training programs. These findings resulted in the following key modifications:

- Focus on the first hour of care: Due to long transport distances and limited access to pediatric referral centers, the curriculum was deliberately restricted to interventions required during initial stabilization rather than definitive hospital management.
- Expansion of the pediatric target population: Initial planning focused primarily on adolescents. Following field observations and stakeholder feedback, younger pediatric patients were incorporated because local providers reported a substantial burden of trauma in children below 12 years of age.
- Adaptation to locally available equipment and medications: Scenario design, SOPs, and skill stations were modified based on inventories provided by SAMU, UPA, and HRSCF. Interventions requiring equipment unavailable in the region were excluded.
- Emphasis on basic, high-impact procedures: Observations revealed limited pediatric equipment availability, variable provider experience, and inconsistent use of standardized protocols. Consequently, the curriculum prioritized ABCDE assessment, hemorrhage control, airway management with supraglottic devices, intraosseous access, pediatric resuscitation, and structured team communication.
- Strengthening of pre-hospital–hospital interface training: Stakeholders repeatedly highlighted communication challenges between SAMU, UPA, and hospital services. As a result, multidisciplinary simulation scenarios and CRM elements were specifically designed to promote structured communication and interprofessional collaboration.
- Train-the-trainer approach: Due to high workforce turnover and limited access to continuing education, local stakeholders recommended establishing a group of long-term employees as future multiplicadores. This resulted in the integration of a dedicated instructor training component.
- Interprofessional training program: The objective was not to certify participants in procedures outside their legal scope of practice but to establish a shared clinical language, common treatment priorities, and coordinated team responses during pediatric trauma care. Accordingly, all participants were introduced to the indications, principles, and practical implications of critical interventions, including needle decompression and intraosseous access, irrespective of profession. This approach was considered particularly relevant in view of the needs assessment findings, which suggested relatively limited experience among physicians and substantial practical experience among nursing personnel.

### 3.2. Needs Assessment (Timepoint 1)

#### 3.2.1. Participant Characteristics

A total of 98 healthcare professionals participated in the needs assessment. The majority were employed in the pre-hospital emergency medical service (SAMU) (*n* = 59; 60.2%), followed by the regional hospital (HRSCF) (n = 16; 16.4%) and the emergency care unit (UPA) (*n* = 9; 9.2%). Additional affiliations were reported by 28 participants (28.6%). As multiple responses were permitted, some participants reported employment at more than one institution.

Regarding professional background, most participants were nursing technicians or nurses (61/98; 62.2%), whereas physicians and paramedics accounted for a smaller proportion (10/98; 10.2%). The remaining participants (26.5%) belonged to other professional groups.

The mean duration of professional experience was 9.7 ± 6.3 years (Table 3).

#### 3.2.2. Knowledge and Confidence in Trauma Management (Adults vs. Pediatric)

Of the 98 healthcare professionals participating in the needs assessment, 66 (67.3%) completed the pediatric trauma management section of the questionnaire. Participants who indicated that they were not involved in the treatment of pediatric patients did not complete this section by design and were therefore not included in the corresponding analyses. Consequently, the baseline confidence analyses reflect only healthcare professionals with direct involvement in pediatric trauma care and should not be generalized to the entire study population.

Overall, 86% of participants reported familiarity with the ABCDE approach, and 77% indicated confidence in its application. Among physicians and paramedics (*n* = 8), agreement with both statements was 100%.

Participants reported significantly lower confidence in managing pediatric trauma compared with adult trauma. Among all respondents, 68% somewhat or strongly agreed that they felt capable of managing complex trauma in adults, whereas only 47% reported this level of confidence for pediatric patients (z = 3.127, *p* = 0.0018). A similar pattern was observed among physicians and paramedics, with 88% expressing confidence in adult trauma management compared with 50% for pediatric cases.

**Figure 2:**
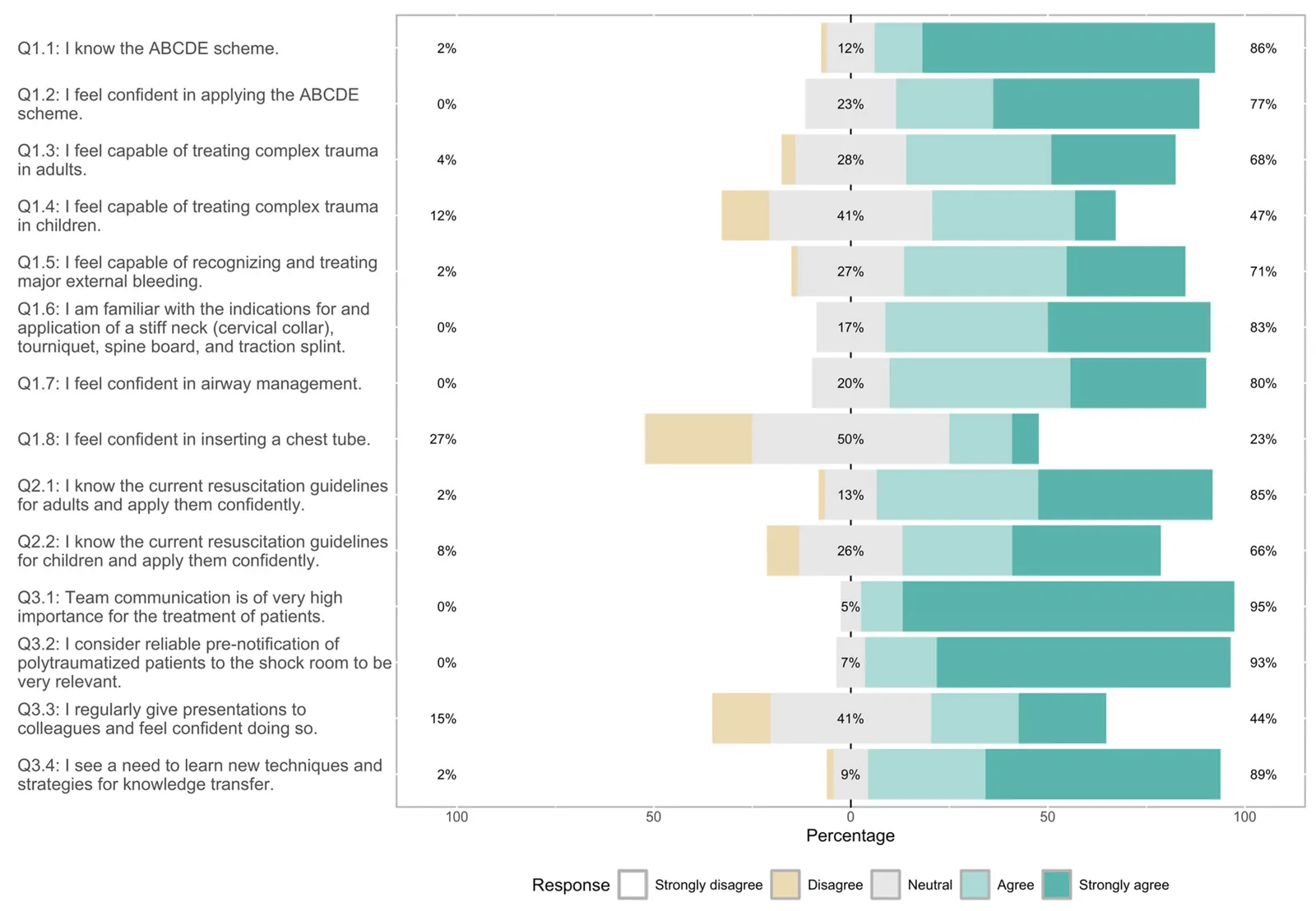
Needs-assessment results: percentages on the right show the sum of (strongly) agree answers, percentages in the middle show neutral responses, and percentages on the left show the sum of (strongly) disagree responses.

Regarding specific competencies, most participants reported confidence in the management of severe external hemorrhage (71%), the indication and application of immobilization and hemorrhage control devices (83%), and airway management (80%). In contrast, confidence in performing chest tube insertion was markedly lower, reported by only 23% of all participants and 38% of physicians and paramedics (z = −7.977, *p* < 0.0001). A comparable discrepancy was observed in familiarity with resuscitation guidelines. Confidence in applying adult resuscitation protocols was higher (85%) than for pediatric protocols (66%), resulting in a statistically significant difference (z = 2.805, *p* = 0.005) (Figure 2).

#### 3.2.3. Non-Technical and Teaching Skills

Participants rated non-technical skills, particularly team communication, as highly relevant. A total of 95% somewhat or strongly agreed that effective communication is critical for patient care, and 93% emphasized the importance of reliable pre-arrival notification of polytraumatized patients.

In contrast, deficits were identified in knowledge dissemination. Only 44% of participants reported that they regularly deliver presentations to colleagues and feel confident in doing so. Notably, 89% expressed a need for additional training in effective teaching and knowledge transfer strategies (Figure 2).

### 3.3. Implementation Phase Evaluation (Timepoint 2)

Feedback was obtained from 38 Brazilian course participants and all four German instructors following completion of the course. Of the 42 enrolled participants, four were excluded from the analysis because of incomplete questionnaires, resulting in complete datasets from 38 participants and all instructors.

Overall acceptance of the course was high, with a 100% recommendation rate among participants (38/38).

#### 3.3.1. Course Evaluation by the Brazilian Participants

Approximately half of the participants rated both the course duration (52.6%) and the balance between theoretical and practical components (47.4%) as appropriate. However, the remaining participants indicated a need for increased practical training and a longer course duration (Table 4).

All participants, including eight future instructors (multiplicadores), agreed that the course was logically structured and effectively conveyed essential knowledge and skills. Feedback on lectures and teaching materials was consistently positive: 94–100% of participants rated sessions on the ABCDE approach and Crew Resource Management (CRM) as relevant and informative and confirmed that case scenarios reflected realistic clinical situations.

**Figure 3:**
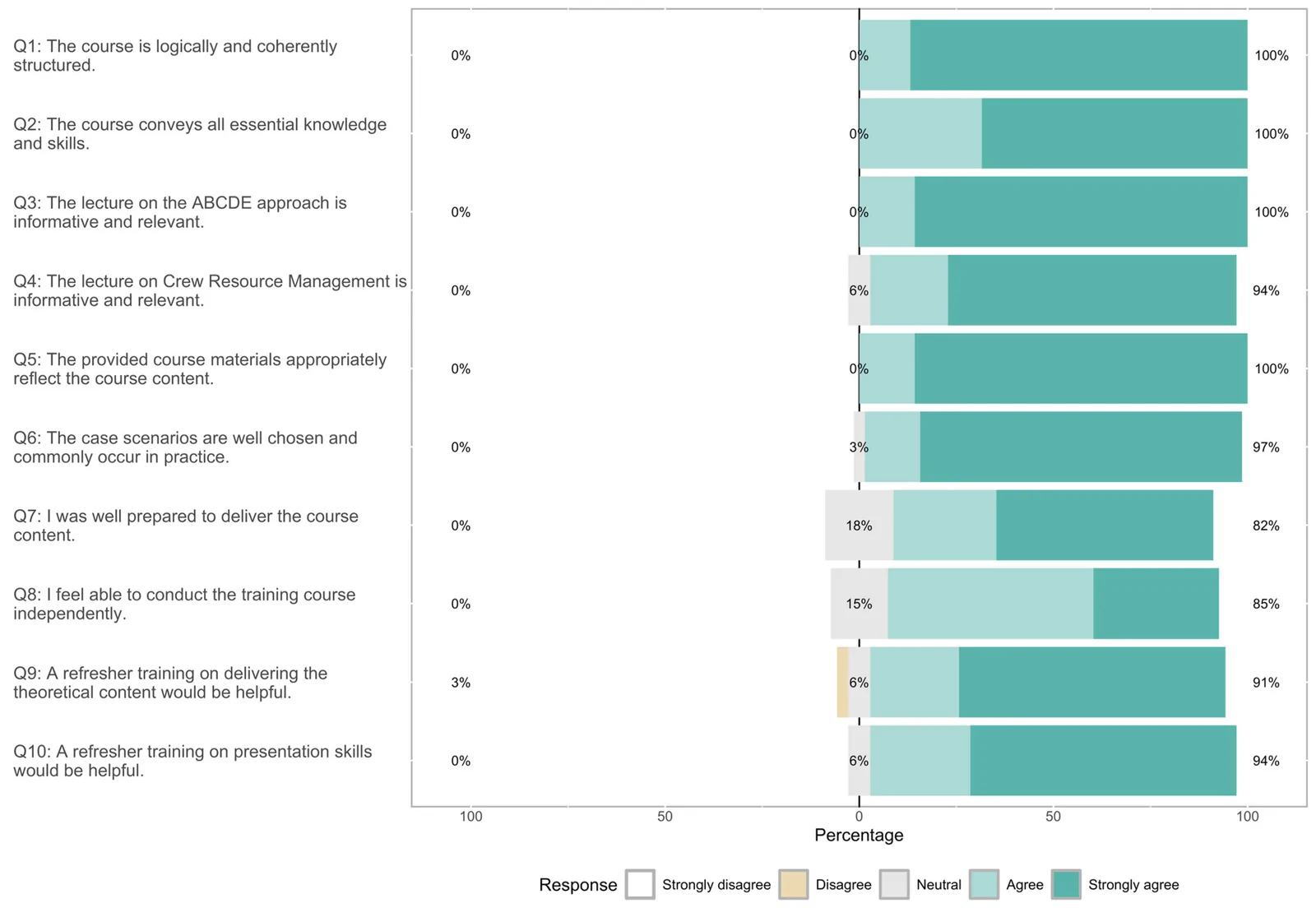
Evaluation of the course content by Brazilian participants: percentages on the right show the sum of (strongly) agree answers, percentages in the middle show neutral responses, and percentages on the left show the sum of (strongly) disagree responses.

Regarding independent course delivery, 82% of participants felt adequately prepared in terms of content, and 85% reported confidence in conducting the course themselves. Nevertheless, over 90% expressed a need for follow-up training, particularly to strengthen theoretical knowledge and teaching competencies (Figure 3).

#### 3.3.2. Course Evaluation by the German Instructor Team

In the initial section of the questionnaire, the four instructors evaluated course structure and organization. Two instructors (50%) recommended increasing the proportion of practical training, while all instructors unanimously supported extending the overall course duration (see Table 4).

Assessment of participants’ prior knowledge was heterogeneous, with 25% somewhat disagreeing, 25% remaining neutral, and 50% somewhat agreeing that participants possessed sufficient baseline knowledge to follow the course. Three out of four instructors agreed that translation did not result in any relevant loss of information and allowed for smooth delivery of the content. All instructors considered the training materials appropriate for achieving the course objectives.

In the second part of the survey, instructors evaluated the lectures and training materials. Three of four instructors agreed that the lectures were appropriate in level and relevance. All instructors confirmed that the course materials accurately reflected the teaching content, that the pediatric skill stations were suitable for the participants’ level of experience, and that the case scenarios were appropriately designed in terms of realism and difficulty.

**Figure 4:**
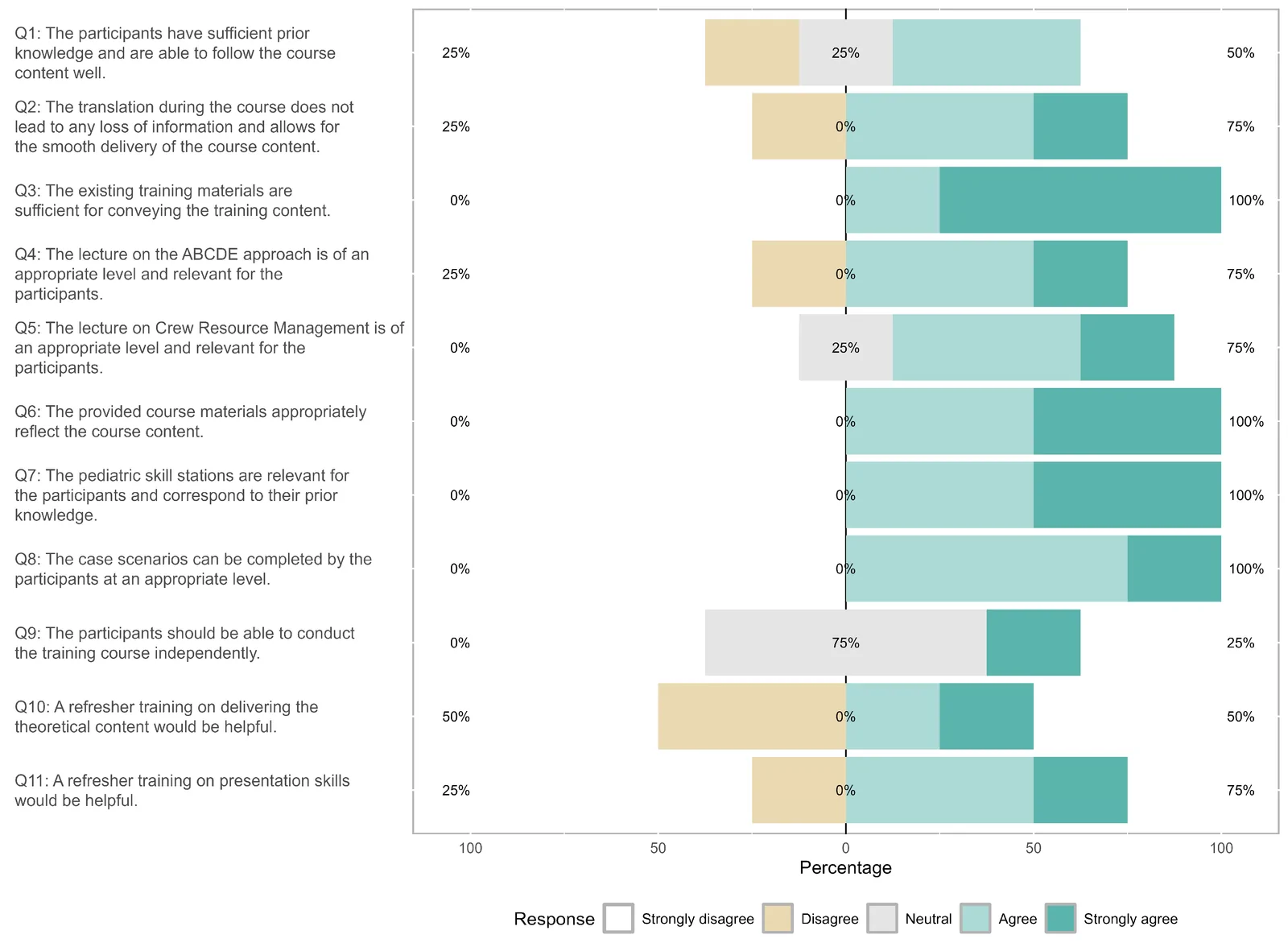
Evaluation of the course content by the German instructor team: percentages on the right show the sum of (strongly) agree answers, percentages in the middle show neutral responses, and percentages on the left show the sum of (strongly) disagree responses.

In contrast, responses regarding participants’ readiness for independent course delivery were more variable. Notably, only one instructor agreed that participants were capable of independently conducting the training. At the same time, there was strong agreement regarding the need for follow-up training, particularly to consolidate theoretical knowledge and to further develop instructional and presentation skills (Figure 4).

## 4. Discussion

### 4.1. Self-Perceived Preparedness Versus Observed Competence

A key finding of this study was the discrepancy between participants’ self-reported preparedness and the more cautious assessment provided by the German instructor team. While more than 80% of participants reported feeling capable of independently delivering the course, only a minority of instructors considered the participants sufficiently prepared to do so. This divergence suggests that self-perceived competence may overestimate actual instructional and clinical proficiency, particularly in newly acquired skills and in educational settings where formal competency-based assessment is uncommon. A possible explanation is the Dunning–Kruger effect, whereby individuals with limited experience or competence tend to overestimate their own abilities due to difficulties in accurately self-assessing their performance [15].

A similar pattern emerged during practical training sessions, where notable discrepancies were observed between self-assessment and actual performance. Deficits were particularly evident in the correct application of tourniquets, airway device placement, and weight-adjusted medication dosing. These findings indicate that familiarity with trauma concepts and high levels of self-confidence do not necessarily translate into procedural competence. The observation is consistent with educational research demonstrating that self-efficacy is an imperfect surrogate for actual performance and should therefore be complemented by objective assessment methods [16].

The discrepancy was further reflected in participants’ high reported familiarity with the ABCDE approach, contrasted with lower confidence in its practical application and by instructor observations during simulation exercises. Together, these findings suggest that conceptual knowledge and self-reported confidence do not necessarily translate into procedural competence or instructional readiness. This discrepancy likely reflects several factors, including limited previous exposure to competency-based assessment, the absence of formal instructor training, and the tendency for high-fidelity simulation courses to enhance learners’ self-efficacy more rapidly than observable teaching competence. Similar discrepancies between perceived and objectively assessed competence have been described in simulation-based medical education [16].

These findings highlight the value of simulation-based education not only for knowledge acquisition but also for objective skills assessment, structured feedback, and the identification of competency gaps that may remain undetected through self-report measures alone. They should also be interpreted within the framework of the Kirkpatrick model of training evaluation [17]. The present study primarily assessed participants’ reactions, acceptance, and perceived learning outcomes (Kirkpatrick Level 1 and selected aspects of Level 2). Objective measures of competence, behavioral change in clinical practice (Level 3), and patient-related outcomes (Level 4) were not evaluated. Consequently, the findings should be interpreted as evidence of feasibility, acceptance, and perceived preparedness rather than effectiveness in improving clinical performance or patient outcomes.

From an implementation perspective, this discrepancy represents an important lesson for international simulation-based educational programs employing a train-the-trainer approach. Self-reported confidence should not be interpreted as evidence of instructional competence. Instead, structured competency assessment, supervised teaching, and progressive instructor development should be regarded as essential components of sustainable faculty development and international knowledge transfer initiatives.

### 4.2. Limitations

Methodological limitations must therefore be acknowledged.

First, the primary outcomes relied on self-reported measures rather than objective performance metrics. Second, only non-validated questionnaires were used, which limits the generalizability of the findings. Third, the sample size was limited and no formal competency assessment was conducted. Finally, no long-term follow-up was performed to evaluate knowledge retention, behavioral change, or clinical outcomes, and the potential influence of social desirability bias on participants’ responses cannot be excluded.

Future studies should therefore incorporate validated educational and psychometric assessment tools, objective performance measures, structured competency testing, and longitudinal outcome evaluation to determine whether the observed improvements in perceived preparedness translate into sustained changes in clinical practice and patient care.

### 4.3. Trauma Management in High-Income Countries

Implementation of protocolized trauma team systems, including established procedures and coordinated multidisciplinary actions, has been shown to increase survival rates, reduce morbidity, and shorten critical decision-making intervals in trauma patients [18]. Organized trauma systems and designated trauma centers characterized by standardized infrastructure, equipment, and training are associated with improved survival and reduced mortality, especially for patients with major trauma [19]. The introduction of trauma teams, adherence to established trauma protocols and their training, and use of SOPs has independently improved patient outcomes and team performance [20]. Emergency departments with high pediatric readiness defined by consistent protocols, appropriate equipment, trained personnel, and robust infrastructure are associated with significantly improved survival and outcomes for injured children [21,22].

### 4.4. Trauma Management in Low- and Middle-Income Countries

Mortality rates for pediatric trauma in LMICs are double those in high-income countries [8]. A systematic review of trauma care systems in LMICs found a lack of trained personnel, protocolized care, and adaptation of treatment strategies to local resources, thus highlighting the need for standardized, locally adapted trauma care protocols to inform system planning and improve outcomes [10]. Existing pediatric trauma outcome tools are suboptimal for LMICs due to reliance on resources often unavailable in these settings [23]. Trauma training programs in LMICs are not widespread, although context-specific trauma education and protocols are critical for sustainable improvements in pediatric trauma care [12,13].

Significant differences in injury mechanisms and outcomes between LMICs and high-income countries have been highlighted, and establishing trauma registries to inform local policy and implementing SOPs to improve care are recommended [24].

This study evaluated the hypothesis that a pediatric trauma training concept derived from high-income emergency care systems can be effectively adapted to a resource-limited setting. In line with the existing literature, our findings confirm substantial gaps in pediatric trauma care in LMICs, particularly in terms of clinical exposure, qualifications, practical skills, structured approaches, and non-technical competencies. The deliberate focus on the first hour of care and on interventions feasible within resource-constrained environments reflected the clinical realities in the Serra da Capivara region and likely contributed to the high acceptance and perceived relevance of the course content.

Future complementary training formats should additionally address structured in-hospital pediatric emergency care up to definitive transfer to an appropriate tertiary trauma center, as relevant deficits in this area were also identified by the authors. Integration of standardized nursing surveillance systems and escalation metrics may further enable evaluation of the impact of training on clinically meaningful outcomes and represents an important area for future research [25].

### 4.5. Cultural Gaps

Early in the conception phase, relevant epidemiological and structural differences between the two healthcare systems became apparent, particularly with regard to injury incidence, injury patterns and severity, patient age distribution, and the considerable distances between pre-hospital and in-hospital care facilities and available resources. These findings are consistent with published data describing a younger and more severely injured trauma population in Brazil, as well as longer pre-hospital transport times [2,4].

During course implementation, several organizational and contextual factors influenced course delivery. Despite the condensed one-day format, some participants arrived late, some breaks were occasionally prolonged, and a minor proportion of participants did not return after lunch. While no formal assessment of the underlying reasons was conducted, these observations may partly reflect the multiple employment arrangements commonly reported among healthcare professionals in Brazil [7].

Although participants rated team communication as highly relevant in the needs assessment, the implementation of selected Crew Resource Management (CRM) principles, such as “speak up” and “listen up”, appeared challenging in some training scenarios. Observations during simulation exercises suggested that professional role perceptions and hierarchical structures may have influenced communication patterns. Similar challenges have been described in previous studies, which have reported associations between high power distance, status asymmetry, and reduced team communication, error reporting, and collaborative decision-making [26,27]. Consequently, educational interventions targeting non-technical skills may benefit from being accompanied by broader organizational efforts that promote psychologically safe communication while preserving clear professional responsibilities and decision-making structures [28,29].

Given the high staff turnover among physicians and the substantial clinical experience reported among nursing personnel in the needs assessment, alternative team leadership models may warrant further investigation. In particular, nurse-led code systems have shown promising results in select settings in Brazil, although current undergraduate nursing curricula may not yet consistently prepare nurses for such responsibilities [30].

Future iterations may additionally benefit from local faculty development, structured debriefing training, leadership engagement, and institutional support for psychologically safe communication.

The anticipated impact of language barriers was less pronounced than expected. This may be attributable to the continuous involvement of bilingual facilitators, including a Portuguese-speaking physician within the German instructor team and the local ProBrasil project coordinator. Their participation likely facilitated communication throughout both the conception and implementation phases and highlights the importance of local partnerships in international educational initiatives, as well as the consideration of intercultural aspects in the planning and execution of international studies and projects.

The needs assessment identified constrained confidence in the management of pediatric patients, particularly regarding invasive procedures and resuscitation. This finding is consistent with previous evidence indicating insufficient exposure and training in high-risk, low-frequency scenarios among young Brazilian physicians working in pre-hospital emergency care [7]. Similar challenges are also recognized in high-income settings such as Germany, where even experienced pre-hospital teams rarely encounter critically ill or injured children, resulting in a persistent need for targeted training in invasive skills such as airway management and vascular access [31]. Accordingly, these identified gaps were explicitly addressed in the curriculum design.

### 4.6. The Interface Between Pre-Hospital and In-Hospital Systems

The need for an improved handover interface was reflected in strong participant support for a standardized pre-arrival notification system between SAMU and UPA for pediatric emergencies. However, this contrasts with the observed clinical reality, where no standardized system exists and communication is often informal, inconsistent, or absent. This structural deficit in the integration of emergency medical services and emergency departments in Brazil has been previously described and highlights the need for standardized communication and handover processes [32]. While the training increased awareness of this issue, structural barriers to implementation remain and cannot be addressed by educational interventions alone. This represents an important opportunity for system-level improvements and the development of appropriate technological solutions.

### 4.7. Training Material Implications

Training for suspected tension pneumothorax was limited to needle decompression in the Monaldi position, in accordance with international guidelines [14]. More advanced procedures, such as chest tube insertion, were excluded due to high costs and the limited availability of suitable task trainers. Alternatives such as animal tissue –based training were not implemented due to ethical considerations, local contextual constraints, and climate-related limitations affecting material storage and durability. These constraints highlight a broader lack of affordable, context-appropriate simulation tools for invasive pediatric procedures and limit the range of skills that can be realistically trained in resource-limited settings.

Logistical and economic factors also had a substantial impact on project implementation. Global supply chain disruptions, marked increases in the cost of simulation equipment and consumables, and complex Brazilian customs procedures represented significant barriers to timely material delivery. These challenges demonstrate that, beyond curriculum design, administrative and logistical conditions critically influence the scalability of simulation-based training programs in resource-constrained environments and underscore the importance of experienced local and international partners, such as the ProBrasil coordination network.

### 4.8. Simulation-Based Education and Sustainability of the Train-the-Trainer Approach

Simulation-based team training and skill stations formed a central component of the PRACTICE course, enabling the integration of theoretical knowledge with practical skills in realistic, high-stress scenarios. Simulation-based education has consistently been associated with improvements in both technical and non-technical competencies [33], a finding that is supported by the present results in a resource-limited setting [34]. Structured debriefings facilitated reflective learning and reinforced the correct application of trauma algorithms, procedural skills, and team-based principles [35].

As previously mentioned, the integration of Crew Resource Management (CRM) addressed non-technical competencies such as communication, situational awareness, and task management, which were identified as highly relevant during the needs assessment. Given that human factors and communication failures are major contributors to adverse events in trauma care [36], the incorporation of CRM represents a key strength of the course design. Although the extent to which CRM training alone can overcome deeply rooted cultural and organizational barriers remains uncertain, it provided a structured and culturally neutral framework for discussing communication failures and team dynamics without attributing blame, making it a pragmatic entry point for non-technical skills training in hierarchical healthcare systems.

A central objective of the PRACTICE project was to ensure sustainability beyond the initial implementation phase. Early and continuous engagement of local stakeholders, combined with the deliberate selection of long-term staff as future multiplicadores, promoted local ownership, acceptance, and identification with the training program. While participants generally evaluated the course positively and reported high levels of preparedness, both German instructors and Brazilian multiplicadores expressed concerns regarding the adequacy of a single instructor-training day followed by two supervised course deliveries. This observation aligns with the discrepancy between participants’ self-reported readiness and the more cautious assessment by instructors discussed above.

Although participants expressed a preference for a multi-day course format, a one-day structure was selected to ensure feasibility within local operational constraints. Nevertheless, the findings suggest that sustainable implementation will likely require additional instructor development, repeated supervised teaching cycles, and periodic refresher training. Whether a single training cycle is sufficient to establish a durable and self-sustaining training system in a setting characterized by high staff turnover and limited access to continuing professional education remains uncertain and should be evaluated in future studies.

While no formal longitudinal evaluation protocol was established as part of the present study, sustainability was actively supported beyond the initial implementation phase. During the subsequent two years of the PRACTICE project period, three follow-up site visits were conducted to observe locally delivered courses, followed by structured debriefings providing individualized feedback and instructor coaching. In addition, a low-threshold communication channel was maintained through a dedicated messaging group, enabling continuous exchange between the Brazilian multiplicadores and the German faculty. The long-term outcomes of this implementation and follow-up phase are currently being evaluated and will be reported separately.

## 5. Conclusions

We present a one-day pediatric trauma care training program for low-resourced, remote regions of Brazil. The program was generally supported, although additional procedural training cycles could enhance the participants’ satisfaction with the program.

Despite these challenges, the development and integration of local instructors represent a promising strategy for the improvement of pediatric trauma care in settings characterized by limited medical resources, high staff turnover, and restricted access to formal continuing education. Future studies should examine the long-term impact of the measures implemented.

## Figures and Tables

**Table 1:** Overview of project milestones and timeline.

Timepoint	Project Activity
August 2023	Project initiation
September 2023	Start of conception phase
9–17 October 2023	Field visit in Piauí/Brazil
November 2023	Formal retrospective funding approval
January 2024	Needs assessment
February 2024	Conception phase completed and Start of implementation phase
17–19 February 2024	On-site train-the-trainer course
17–28 February 2024	Implementation phase evaluation
29 February 2024	Additional needs assessment at the local emergency medicine congress in Teresina, Piauí
14 March 2024	Implementation phase completed
April 2024	Final course concept delivered to local instructors (*multiplicadores*)

**Table 2:** Course schedule, including duration, number of participants, structure, content, and group assignments.

Key Features			
Format		One-day training course (8 h total, incl. 1 h of breaks)	
Participants		10–12 per unit	
Trainers		1 per 4 participants	
Curriculum			
Period	Content	Location	Groups
0.75 h	Introduction Welcome, introduction of participants, expectations and apprehensions	Lecture room	All
0.75 h	Lectures ABCDE/PAT Approach Crew-Resource-Management	Lecture room	All
2 h	3 Pediatric Skills stations A—cervical spine immobilization, airway management B—assisted and controlled manual ventilation, chest decompression C—trauma resuscitation, vascular access, hemorrhage control D—Glasgow Coma Scale, pupils E—trauma examination, immobilization, pelvic sling, wound management	Large room suitable for 3 tables with materials, 1 table per skill station	3 groups, 3–4 participants per group
3 h	Case scenarios on severe pediatric trauma (0.75 h each) Motorcycle accident Trauma resuscitation Car accident (pedestrian with severe thoracic-abdominal injuries) Followed by each case scenario, debriefing with all participants in lecture room	Response site and Emergency department -setup	3 groups, 1 per scenario In each case scenario, 3–4 active participants and passive spectators
0.5 h	Final round Self-assessment test, evaluation Feedback, farewell	Lecture room	All

**Table 3:** Needs assessment, sample description, *N* = 98.

Employer, *n* (%) *	
SAMU	59 (60.2)
HRSCF	16 (16.4)
UPA	9 (9.2)
Other	28 (28.6)
Professional group, *n* (%)	
Nursing technician	24 (24.5)
Nurse	37 (37.8)
Physician	8 (8.2)
Paramedic	2 (2.0)
Other	26 (26.5)
Work experience in years, mean ± sd (range)	9.7 ± 6.3 (0–25)

Abbreviations: *N*—number of samples; *n*—number of subjects; mean—arithmetic mean; sd—standard deviation; * multiple answers possible. One participant did not report profession.

**Table 4:** Evaluation of the duration and content of the PRACTICE course by Brazilian participants and instructors.

	Role	
	Participant (*N* = 38)	Instructor (*N* = 4)
Duration of training, *n* (%)		
Way too long	0 (0.0)	0 (0.0)
Too long	0 (0.0)	0 (0.0)
Exactly right	18 (47.4)	0 (0.0)
Too short	9 (23.7)	4 (100.0)
Way too short	11 (28.9)	0 (0.0)
Theoretical/practical content, *n* (%)		
Much more theory	0 (0.0)	0 (0.0)
More theory	0 (0.0)	0 (0.0)
Exactly right	20 (52.6)	2 (50.0)
More practical	10 (26.3)	2 (50.0)
Much more practical	8 (21.1)	0 (0.0)

Abbreviations: *N*—number of sample; *n*—number of subjects.

## Data Availability

Further information and reasonable requests for resources should be directed to and will be fulfilled by the first author, Jakob Olbrich (jakob.olbrich@ukb.de). No new code was generated during the course of this study. Any additional information required to reanalyze the data reported in this paper is available from the first author upon request.

## Abbreviations

The following abbreviations are used in this manuscript:

ABCDE	Airway, Breathing, Circulation, Disability, Exposure
AGREE II	Appraisal of Guidelines for Research and Evaluation II
ATLS	Advanced Trauma Life Support
BLS	Basic Life Support
CBMPI	Corpo de Bombeiros Militar do Piauí (Military Fire Brigade of the State)
CRM	Crew Resource Management
DRKS	Deutsches Register Klinischer Studien (German Clinical Trials Register)
ED	Emergency department
GDPR	General Data Protection Regulation
GIZ	Gesellschaft für Internationale Zusammenarbeit (German Agency for International Cooperation)
HRSCF	Hospital Regional Senador Cândido Ferraz
LMICs	Low- and middle-income countries
mean	Arithmetic mean
*n*	Number of subjects
*N*	Sample size
NGO	Non-Governmental Organization
OSCE	Objective structured clinical examination
PALS	Pediatric Advanced Life Support
PHTLS	Pre-Hospital Trauma Life Support
PRACTICE	PReventing And Customizing Treatment of road traffic associated Injuries in ChildrEn; project and course name
SAMU	Serviço de Atendimento Móvel de Urgência (Mobile Emergency Medical Service)
sd	Standard deviation
SENAC	Serviço Nacional de Aprendizagem Comercial
SESAPI	Secretaria de Estado da Saúde do Piauí (State Health Secretariat of Piauí)
SOPs	Standard operating procedures
TECC	Tactical Emergency Casualty Care
UPA	Unidade de Pronto Atendimento (Emergency Department)
UESPI	Universidade Estadual do Piauí (State University of Piauí)

## References

[1] Peden M., Oyegbite K., Ozanne-Smith J., Hyder A.A., Branche C., Rahman A.F., Rivara F., Bartolomeos K., World Report on Child Injury Prevention (2008). WHO Guidelines Approved by the Guidelines Review Committee.

[2] Viana S.W., Gerk A., Schlindwein S.S., Marrazzo E., Feres B., Ribeiro L., Carroll M., Mooney D.P., Schnitman G., Camargo C.P. (2025). Public Health System Expenditure on Motor Vehicle Collisions in Brazil: An Ecological Study. Acta Cir. Bras..

[3] (2023). Global Status Report on Road Safety 2023.

[4] Martins E.T., Boing A.F., Peres M.A. (2013). Motorcycle accident mortality time trends in Brazil, 1996–2009. Rev. Saude Publica.

[5] Costa R.E.A.R.D., Visgueira A.R.L., Sousa R.F.V.D., Monteiro K.J.L., Horta M.A.P., Oliveira B.F.A.D. (2023). Tendência de Indicadores de Morbimortalidade Por Doenças Diarreicas Agudas Em Menores de Cinco Anos No Estado Do Piauí (2000–2019). Rev. Bras. Saúde Materno Infant..

[6] Botelho F., Truche P., Mooney D.P., Caddell L., Zimmerman K., Roa L., Alonso N., Bowder A., Drumond D., Abib S.D.C.V. (2020). Pediatric Trauma Primary Survey Performance among Surgical and Non-Surgical Pediatric Providers in a Brazilian Trauma Center. Trauma Surg. Acute Care Open.

[7] Tallo F.S., de Campos Vieira Abib S., Baitello A.L., Lopes R.D. (2014). An Evaluation of the Professional, Social and Demographic Profile and Quality of Life of Physicians Working at the Pre-hospital Emergency Medical System (SAMU) in Brazil. Clinics.

[8] Ali A.E., Sharma S., Elebute O.A., Ademuyiwa A., Mashavave N.Z., Chitnis M., Abib S., Wahid F.N. (2023). Trauma and Sexual Abuse in Children—Epidemiology, Challenges, Management Strategies and Prevention in Lower- and Middle-Income Countries. Semin. Pediatr. Surg..

[9] Amado V., Couto M.T., Filipe M., Möller J., Wallis L., Laflamme L. (2023). Assessment of Critical Resource Gaps in Pediatric Injury Care in Mozambique’s Four Largest Hospitals. PLoS ONE.

[10] Reynolds T.A., Stewart B., Drewett I., Salerno S., Sawe H.R., Toroyan T., Mock C. (2017). The Impact of Trauma Care Systems in Low- and Middle-Income Countries. Annu. Rev. Public Health.

[11] Pai P.K., Klinkner D.B. (2022). Pediatric Trauma in the Rural and Low Resourced Communities. Semin. Pediatr. Surg..

[12] Livergant R.J., Demetrick S., Cravetchi X., Kung J.Y., Joos E., Hawes H.G., Saleh A. (2021). Trauma Training Courses and Programs in Low- and Lower Middle-Income Countries: A Scoping Review. World J. Surg..

[13] Pinkham L., Botelho F., Khan M., Guadagno E., Poenaru D. (2022). Teaching Trauma in Resource-Limited Settings: A Scoping Review of Pediatric Trauma Courses. World J. Surg..

[14] Freire G.C., Beno S., Yanchar N., Weiss M., Stang A., Stelfox T., Bérubé M., Beaulieu E., Gagnon I.J., Zemek R. (2023). Clinical Practice Guideline Recommendations For Pediatric Multisystem Trauma Care: A Systematic Review. Ann. Surg..

[15] Kruger J., Dunning D. (1999). Unskilled and unaware of it: How difficulties in recognizing one’s own incompetence lead to inflated self-assessments. J. Pers. Soc. Psychol..

[16] Williams D.M., Rhodes R.E. (2016). The Confounded Self-Efficacy Construct: Conceptual Analysis and Recommendations for Future Research. Health Psychol. Rev..

[17] Falletta S. (1998). Evaluating Training Programs: The Four Levels Donald L. Kirkpatrick, Berrett-Koehler Publishers, San Francisco, CA, 1996, 229 pp. Am. J. Eval..

[18] Chang Y.-R., Wang H.-L.C., Wu C., Chen P.-C., Lin K.-L., Fu C.-Y., Lin H.-F. (2026). Protocolized Trauma Team Activations Improve Trauma Patient Outcomes and Decrease Decision-Making Intervals. Am. J. Emerg. Med..

[19] Moore L., Champion H., Tardif P., Kuimi B., O’Reilly G., Leppaniemi A., Cameron P., Palmer C.S., Abu-Zidan F.M., Gabbe B. (2018). Impact of Trauma System Structure on Injury Outcomes: A Systematic Review and Meta-Analysis. World J. Surg..

[20] Cuschieri J., Johnson J.L., Sperry J., West M.A., Moore E.E., Minei J.P., Bankey P.E., Nathens A.B., Cuenca A.G., Efron P.A. (2012). Benchmarking Outcomes in the Critically Injured Trauma Patient and the Effect of Implementing Standard Operating Procedures. Ann. Surg..

[21] Newgard C.D., Lin A., Olson L.M., Cook J.N.B., Gausche-Hill M., Kuppermann N., Goldhaber-Fiebert J.D., Malveau S., Smith M., Dai M. (2021). Evaluation of Emergency Department Pediatric Readiness and Outcomes Among US Trauma Centers. JAMA Pediatr..

[22] Melhado C., Hancock C., Wang H., Eldin M.M., George N., Miller J.A., Remick K.E., Patel B., Yorkgitis B.K., Gray L. (2025). Pediatric Readiness and Trauma Center Access for Children. JAMA Pediatr..

[23] St-Louis E., Séguin J., Roizblatt D., Deckelbaum D.L., Baird R., Razek T. (2017). Systematic Review and Need Assessment of Pediatric Trauma Outcome Benchmarking Tools for Low-Resource Settings. Pediatr. Surg. Int..

[24] Bradshaw C.J., Bandi A.S., Muktar Z., Hasan M.A., Chowdhury T.K., Banu T., Hailemariam M., Ngu F., Croaker D., Bankolé R. (2018). International Study of the Epidemiology of Paediatric Trauma: PAPSA Research Study. World J. Surg..

[25] Cesare M., Nurchis M.C., Damiani G., Cocchieri A., Nursing and Public Health Group (2025). Ranking Nursing Diagnoses by Predictive Relevance for Intensive Care Unit Transfer Risk in Adult and Pediatric Patients: A Machine Learning Approach with Random Forest. Healthcare.

[26] Greer L.L., De Jong B.A., Schouten M.E., Dannals J.E. (2018). Why and When Hierarchy Impacts Team Effectiveness: A Meta-Analytic Integration. J. Appl. Psychol..

[27] Stevens E.L., Hulme A., Salmon P.M. (2021). The Impact of Power on Health Care Team Performance and Patient Safety: A Review of the Literature. Ergonomics.

[28] Hughes M.K., Benenson R.S., Krichten A.E., Clancy K.D., Ryan J.P., Hammond C. (2014). A Crew Resource Management Program Tailored to Trauma Resuscitation Improves Team Behavior and Communication. J. Am. Coll. Surg..

[29] Kehoe A., Ellawala A., Karunaratne D., Tiffin P.A., Crampton P.E.S. (2026). Effective Teamwork within Healthcare–Let’s Finally Make It Happen! A Realist Evaluation. Med. Teach..

[30] Magnago C., Pierantoni C.R. (2021). Situational Analysis and Reflections on the Introduction of Advanced Practice Nurses in Brazilian Primary Healthcare. Hum. Resour. Health.

[31] Mockler S., Metelmann C., Metelmann B., Thies K.C. (2023). Prevalence and Severity of Pediatric Emergencies in a German Helicopter Emergency Service: Implications for Training and Service Configuration. Eur. J. Pediatr..

[32] Souza M.M.D., Xavier A.C., Araújo C.A.R., Pereira E.R., Duarte S.D.C.M., Valladares Broca P. (2020). Communication between Pre-Hospital and Intra-Hospital Emergency Medical Services: Literature Review. Rev. Bras. Enferm..

[33] Elendu C., Amaechi D.C., Okatta A.U., Amaechi E.C., Elendu T.C., Ezeh C.P., Elendu I.D. (2024). The Impact of Simulation-Based Training in Medical Education: A Review. Medicine.

[34] Glomb N.W., Rus M.C., Kosoko A.A., Saha S., Murphy K., Doughty C.B., Galapi C., Laba B., Shah M.I. (2021). Pediatric Simulation-Based Prehospital Training Course in Botswana. J. Educ. Teach. Emerg. Med..

[35] Albar I.A., Sw Menaldi S.L., Findyartini A. (2025). Role of Debriefing in Trauma Simulation Training to Improve Trauma Team Performance in Emergency Department. EIMJ.

[36] Gloor S., Quante M., Lehmann B., Schnüriger B. (2025). Breaking the Silence: Communication Failures Are the Leading Errors Identified in 10-Years of Trauma Morbidity and Mortality Conferences. Eur. J. Trauma. Emerg. Surg..

